# Mahalanobis distance, a novel statistical proxy of homeostasis loss is longitudinally associated with risk of type 2 diabetes

**DOI:** 10.1016/j.ebiom.2021.103550

**Published:** 2021-08-20

**Authors:** Jose L. Flores-Guerrero, Marco A. Grzegorczyk, Margery A. Connelly, Erwin Garcia, Gerjan Navis, Robin P.F. Dullaart, Stephan J.L. Bakker

**Affiliations:** a Department of Internal Medicine, Division of Nephrology, University of Groningen, University Medical Center Groningen, Hanzeplein 1, Groningen 9713 GZ, the Netherlands; b Johann Bernoulli Institute, University of Groningen, Groningen, the Netherlands; c Laboratory Corporation of America Holdings (Labcorp), Morrisville, North Carolina, USA; d Department of Internal Medicine, Division of Endocrinology, University of Groningen, University Medical Center Groningen, Groningen, the Netherlands

**Keywords:** Type 2 diabetes, Mahalanobis distance, Homeostasis, Dysregulation, Biomarkers

## Abstract

**Background:**

The potential role of individual plasma biomarkers in the pathogenesis of type 2 diabetes (T2D) has been broadly studied, but the impact of biomarkers interaction remains underexplored. Recently, the Mahalanobis distance (MD) of plasma biomarkers has been proposed as a proxy of physiological dysregulation. Here we aimed to investigate whether the MD calculated from circulating biomarkers is prospectively associated with development of T2D.

**Methods:**

We calculated the MD of the Principal Components (PCs) integrating the information of 32 circulating biomarkers (comprising inflammation, glycemic, lipid, microbiome and one-carbon metabolism) measured in 6247 participants of the PREVEND study without T2D at baseline. Cox proportional-hazards regression analyses were performed to study the association of MD with T2D development.

**Findings:**

After a median follow-up of 7·3 years, 312 subjects developed T2D. The overall MD (mean (SD)) was higher in subjects who developed T2D compared to those who did not: 35·65 (26·67) and 30.75 (27·57), respectively (*P* = 0·002). The highest hazard ratio (HR) was obtained using the MD calculated from the first 31 PCs (per 1 log-unit increment) (1·72 (95% CI 1·42,2·07), *P* < 0·001). Such associations remained after the adjustment for age, sex, plasma glucose, parental history of T2D, lipids, blood pressure medication, and BMI (HR_adj_ 1·37 (95% CI 1·11,1·70), *P* = 0·004).

**Interpretation:**

Our results are in line with the premise that MD represents an estimate of homeostasis loss. This study suggests that MD is able to provide information about physiological dysregulation also in the pathogenesis of T2D.

**Funding:**

The Dutch Kidney Foundation (Grant E.033).

**Research in context**

**Evidence before this study**

A large body of evidence suggest that circulating biomarkers are associated with future development of Type 2 diabetes. Nevertheless, most of the studies investigate such biomarkers separately. It has been recently proposed that the Mahalanobis distance of circulating biomarkers can be used as a measure of homeostasis loss that occurs with ageing. Whether this statistical approach could be useful in the study of T2D development remains to be explored.

**Added value of this study**

In the present study, we reveal that the Mahalanobis distance of circulating biomarkers, including lipid metabolism, as well as inflammation and microbiome derived metabolites, provide information about the physiological dysregulation not only in ageing process, as previously reported, but also in the development of T2D.

**Implications of all the available evidence**

The prognostic dysregulation signature represented by the Mahalanobis distance may represent a tool to integrate several biomarkers in the study of homeostasis loss in prospective studies. In addition, given the dynamic nature of the biomarkers analyzed in the present study, this statistical approach could potentially help to elucidate effects of interventions, beyond the assessment of single biomarker measurements.

Alt-text: Unlabelled box

## 1 Introduction

The idea of an internal body regulation mechanism as a cornerstone in health and disease has been around in medicine since the pre-Socratic era [1]. Bernard and Cannon helped to integrate such ideas under the concept of homeostasis, defined as a self-regulating process by which an organism can maintain internal stability while adjusting to changing external conditions [2].

The role of homeostasis in the study of chronic diseases had gained attention over time as they became more prevalent [3]. Amongst the most prevalent chronic diseases, type 2 diabetes (T2D) has become one of the leading causes of morbidity and disability worldwide [4]; consequently, several studies had been performed to understand the underlying mechanism of the homeostasis loss in T2D [3,[5], [6], [7]].

The impact of homeostasis loss in the development of T2D has included the role of the pancreas [8], skeletal muscle [9], adipose tissue [10], and more recently the gut microbiota [11]. Although the interconnection of such systems in health and disease has been recognized, analyzes of their related biomarkers has largely been confined to individual biomarkers to date. For instance, in a study comprising 11,896 subjects from four well characterized prospective cohorts, 113 out of 229 metabolites were associated with risk of T2D development [12]. Several other observational studies have confirmed these findings [13], [14], [15]. Remarkably, the assessment of how the simultaneous variation of such biomarkers among themselves could reflect the loss of homeostasis remains underexplored in the context of T2D development.

Recently, it has been suggested that the analysis of the statistical distances of multivariate probability distributions, using circulating biomarkers, could identify abnormalities in the overall biomarker profile of subjects in relation to the studied population [16]. Particularly, the Mahalanobis distance (MD) calculated from circulating biomarkers has been suggested as a proxy of homeostasis loss, with a higher MD being associated with ageing related outcomes [16]. Therefore, the aim of this study was to investigate whether the MD calculated from circulating biomarkers is longitudinally associated with development of T2D among participants from a large general population-based cohort study.

## 2 Methods

### 2.1 Study design and subjects

The Prevention of Renal and Vascular END-stage Disease (PREVEND) Study is a population-based cohort study in the city of Groningen, The Netherlands. The PREVEND study was designed to prospectively investigate the natural course of increased levels of urinary albumin excretion and its relation to renal and cardiovascular disease in a large cohort drawn from the general population [17]. The design of the PREVEND Study has been described in detail elsewhere [18]. Briefly, from 1997 to 1998, all residents from Groningen, excluding pregnant women and people with type 1 diabetes or T2D using insulin, aged 28–75 years were invited to participate. A total of 40,856 subjects (47.8%) responded the invitation to participate. From this group, 30,890 subjects had a urinary albumin concentration of < 10 mg/L and 9966 subjects had a urinary albumin concentration of ≥ 10 mg/L in their morning urine sample. After exclusion of subjects with type 1 diabetes and pregnant women, all subjects with a urinary albumin concentration of ≥ 10 mg/L (*n* = 7768) and a randomly selected control group with a urinary albumin concentration of < 10 mg/L (*n* = 3395) were invited for further investigations in an outpatient clinic. A total of 8592 individuals completed an extensive examination.

We used data of participants who completed the second screening (*n* = 6894), and were free from T2D at baseline (*n* = 6447) excluding those with insufficient samples for quantification of biomarkers by means of Nuclear Magnetic Resonance (NMR), leaving a cohort of 6247 participants with complete information for the analyzes. Cases of participants lost to follow-up were considered as censored cases. This report follows the Strengthening the Reporting of Observational Studies in Epidemiology (STROBE) reporting guideline (Supplemental Table 1).

### 2.2 Ethics

The study conforms to the ethical guidelines of the 1975 Declaration of Helsinki [19] and was approved by the local ethics committee of the University Medical Center Groningen (approval number: MEC96/01/022). All participants provided written informed consent.

### 2.3 Clinical measurements

During two outpatient visits, baseline data were collected on demographics, lifestyle factors, anthropometric measurements, medical history, parental history of T2D and medication use. Information on medication use was combined with information from a pharmacy-dispensing registry, which had complete information on the drug usage of > 95% of subjects in the PREVEND study. Height and weight were measured in standing position without shoes and heavy outer garments. Body mass index (BMI) was calculated as weight (kg) divided by height squared (meter). Waist circumference was measured as the smallest girth between the rib cage and iliac crest. Systolic and diastolic blood pressure values were measured with an automatic Dinamap XL Model 9300 series device and recorded as the means of the last two recordings of the second visit.

### 2.4 End point of the study

Participants were followed from the date of the baseline center visit until end of follow-up. Incident T2D was established if one or more of the four criteria were met during follow-up: (1) blood glucose ≥ 7·0 mmol/L (126 mg/dL); (2) random sample plasma glucose ≥ 11·1 mmol/L (200 mg/dL); (3) self-report of a physician diagnosis; (4) initiation of glucose lowering medication according to the central pharmacy registry follow-up data, which was completed as of 1 January 2011.

### 2.5 Laboratory measurements

Laboratory measurements were performed at the Central Laboratory of the University Medical Center Groningen. The Netherlands. Venous blood samples were drawn after an overnight fast of at least 8 h, while participants rested for 15 min. Ethylene diamine tetra acetic acid (EDTA) - anticoagulated plasma samples and sera were stored at -80 °C until analysis.

Fasting plasma glucose (FPG) was measured by dry chemistry (Eastman Kodak, Rochester, NY, USA). Insulin was measured with an immunoturbidometric assay (Diazyme Laboratories, Poway, CA, USA). Total cholesterol (TC), triglycerides, and serum creatinine were measured using standard protocols, as described previously [20]. Serum alanine aminotransferase (ALT) and aspartate aminotransferase (AST) were measured using the standardized kinetic method with pyridoxal phosphate activation (Roche Modular P, Roche Diagnostics, Mannheim, Germany). Serum gamma-glutamyl transferase (GGT) was assayed using an enzymatic colorimetric method (Roche Modular P, Roche Diagnostics, Mannheim, Germany). Standardization of ALT, AST and GGT was performed according to the International Federation of Clinical Chemistry guidelines [21], [22], [23]. High-sensitivity C-reactive protein (hs-CRP) albumin and urea concentrations were measured with Roche routine chemistry analyzers (Modular P/Cobas C, Roche Diagnostics, Mannheim, Germany). Urinary albumin was measured by nephelometry (Dade Behring Diagnostic, Marburg, Germany) and urinary albumin excretion (UAE) was determined in two 24 h urine collections with the results being averaged for analysis. Serum creatinine was measured by an enzymatic method on a Roche Modular analyzer (Roche Diagnostics, Mannheim, Germany). Serum ferritin was measured using immunoassay (Roche Diagnostics, Mannheim, Germany). Hemoglobin, hematocrit, and mean corpuscular volume, were measured on a Coulter Counter STKS sample testing system (Coulter Corp) in fresh venous blood according to standard procedures.

Trimethylamine N-oxide (TMAO), betaine, branched chain amino acids (BCAA), GlycA (a pro-inflammatory glycoprotein biomarker), high density lipoprotein (HDL) and ketone body concentrations were measured in EDTA-anticoagulated plasma samples using a Vantera® Clinical Analyzer (LabCorp, Morrisville, NC), a fully automated, high-throughput, 400 MHz NMR spectroscopy platform using spectral deconvolution algorithm as previously described [24], [25], [26], [27], [28], [29], [30]. A detailed description of the NMR biomarker measurements is found in the Supplement.

### 2.6 Statistical analysis

Normality distribution was assessed with the visualization of density plots and Q-Q plots. Variables with a skewed distribution were natural log transformed. Baseline data were presented as the mean (standard deviation, SD) or median (interquartile range, IQR) for continuous variables and percentages for categorical variables. All statistical analyzes were performed with R language for statistical computing software, v. 4·0·3 (2020), (Vienna, Austria) [31].

#### 2.6.1 Mahalanobis distance

We used a set of thirty-two circulating metabolites to calculate the MD. Twenty-three out of the thirty-two biomarkers have already been reported to be useful in the calculation of MD as proxy of homeostasis loss (Supplemental Table 2) [16]. We replaced HDL cholesterol, used in a previous report [16], with seven HDL subspecies that have lately been reported to be differently associated with the risk of T2D [32]. Additionally, BCAA (valine, leucine, isoleucine) were included in the analysis because of their association with T2D [15], which has been shown to be causal [33]. Finally, TMAO and betaine, were included as gut-microbiota derived metabolites, given the recently recognized relationship with the gut microbiome in the context of T2D [34].

To better depict the intervariability of the circulating biomarkers, a Principal Component Analysis (PCA) was performed using the biomarker data, in order to obtain a new set of variables which integrates the information of the 32 biomarkers. Considering that the circulating biomarkers are measured in different units, the input data was standardized in order to have mean equals to zero and variance equals to one before doing PCA. PCA is a dimensionality reduction technique, which comprehends a series of orthogonal linear transformations of the original variables, generating a new set of transformed variables (denominated Principal Components (PCs)). Each PC is a linear combination of all *p* variables, and it is intended that the new set of transformed variables preserves as much as possible of the information contained in the original variables [35].

Starting with the addition of PC1 and PC2, the 32 PCs were added one by one into cumulative sets of PCs that were used to calculate 31 different MDs.

The MD is a multivariate distance measure related to the familiar Euclidean Distance; yet, it provides two further benefits. Firstly, it is scale invariant, meaning that the differences in the unit measurements of the diverse biomarkers do not bias the analysis. Secondly, it includes the correlations between the covariates, allowing to capture the information not only for the difference in one variable, but rather, the differences among a set of variables [36]. The MD is defined as: *MD* (*x_i_*, *x_j_*) = [(*x_i_*−*x_j_*)*^T^****S***^−1^(*x_i_*−*x_j_*)]^1/2^ where x_i_ is the *i*th row of the (*n* × *p*) covariate matrix ***X***, with *n* subjects in the rows and *p* covariates in the columns, and ***S*** is the (*p* × *p*) covariance matrix of ***X*** [36].

#### 2.6.2 Survival analysis

Time-to-event Cox proportional hazards models were used to compute hazard ratios (HRs) and 95% CI of T2D development risk, using the MDs calculated from subsets of the PCs. In order to evaluate the potential overfitting of the different models, the Bayesian Information Criterion (BIC) and the Akaike Information Criterion (AIC) were computed for the MDs that contained different subsets of the PCs. Given the fact that mean and median of the MD increases with a larger number the variables included in its calculation, we further calculated the Relative Risk difference over 95% of the observed MD distribution; this was calculated by subtracting the risk of being in the 97·5th percentile of MD relative to the 2·5th percentile, this method was previously reported to be appropriate to compare different MDs [37]. HRs were calculated per 1-unit increase in the log scale. HRs were adjusted for age and sex, BMI (or waist circumference), plasma glucose, lipid lowering medication and anti-hypertensive medication. The Cox proportional hazard assumption was tested through the evaluation of independence between scaled Schoenfeld residuals with time for each variable and for every model as a whole; this assumption was met, with no indication for a violation [38].

To further evaluate the performance of the MD to improve the T2D risk reclassification, two risk prediction models were fitted: The first model included the clinical variables used in the FINDRISC T2D risk score, which has been reported as a reasonably good predictor of incident T2D in the Netherlands [39] (age, family history of T2D, BMI, waist circumference, hypertension and FPG). The second model included the MD in addition to the variables above mentioned. Using predefined risk categories of T2D development (< 10%), intermediate (10 to 20%), and high ( ≥ 20%) [40], reclassification was assessed using the categorical net reclassification improvement (NRI) approach; additionally, a category-free NRI was also computed [41].

### 2.7 Role of the funding source

The funders did not have any role in study design, data collection, data analyzes, interpretation, or writing of report.

## 3 Results

### 3.1 Clinical characteristics at baseline

A total of 6247 participants of the PREVEND cohort were included in this study. Among the participants, 3089 (49·4%) were men, and the mean age of the population was 53·2 (11·0) years. Participant characteristics at baseline are shown in Table 1. During a median follow-up of 7·3 (IQR 6·1–7·3) years, a total of 312 participants developed T2D. Participants who developed T2D during the follow-up, were more likely to be men and to be older, and were more likely to have a family history of diabetes when compared to people who did not develop T2D. Likewise, those who developed T2D, presented a higher BMI, waist circumference and blood pressure. Among T2D developers it was more common to have a history of CVD and parental history of T2D; those participants also used antihypertensive medications and lipid-lowering drugs more frequently. There was no difference in terms of a history of cancer, smoking or alcohol consumption.

**Table 1:** Baseline characteristics of 6247 participants of the PREVEND prospective cohort.

Variable	Total(*N* = 6247)	Incident T2D(*N* = 312)	No Incident T2D (*N* = 5935)	*P* value
Men, (%)	3089 (49·4%)	196 (62·8%)	2893 (48·7%)	< 0·001
Age, years	53·18 (11·94)	57·47 (9·97)	52·96 (12·00)	< 0·001
BMI, kg/m^2^	26·50 (4·22)	29·96 (4·67)	26·32 (4·12)	< 0·001
Waist circumference, cm	91·55 (12·56)	102·27 (12·38)	90·99 (12·32)	< 0·001
SBP, mmHg	125·64 (18·60)	136·84 (21·05)	125·05 (18·27)	< 0·001
DBP, mmHg	73·24 (9·08)	77·60 (9·39)	73·01 (9·01)	< 0·001
History of Cancer, (%)	279 (4·5%)	12 (3·9%)	267 (4·5%)	0·68
History of CVD, (%)	231 (3·7%)	22 (7·1%)	209 (3·5%)	0·001
Parental history of T2D, (%)	898 (14·9%)	87 (29·3%)	811 (14·1%)	< 0·001
Smoking status, (%)				0·47
never	1788 (28·6%)	81 (26·0%)	1707 (28·8%)	
former	2626 (42·0%)	140 (44·9%)	2486 (41·9%)	
current < 6 cig/day	284 (4·5%)	14 (4·5%)	270 (4·5%)	
current 6–20 cig/day	1231 (19·7%)	58 (18·6%)	1173 (19·8%)	
current > 20 cig/day	243 (3·9%)	17 (5·4%)	226 (3·8%)	
Alcohol consumption, (%)				
No, almost never	1512 (24·4%)	89 (28·6%)	1423 (24·2%)	0·14
1–4 drinks/month	1058 (17·1%)	49 (15·8%)	1009 (17·2%)	
2–7 drinks/week	1976 (31·9%)	88 (28·3%)	1888 (32·1%)	
1–3 drinks/day	1380 (22·3%)	66 (21·2%)	1314 (22·4%)	
4 or more drinks/day	264 (4·3%)	19 (6·1%)	245 (4·2%)	
Lipid-lowering drugs, (%)	446 (7·1%)	50 (16·0%)	396 (6·7%)	< 0·001
Antihypertensive drugs, (%)	1130 (18·1%)	115 (36·9%)	1015 (17·1%)	< 0·001
Glucose, mmol/L	4·70 (4·40, 5·20)	5·80 (5·20, 6·20)	4·70 (4·40, 5·20)	< 0·001
eGFR, mL/min/1·73 m^2^	92·45 (16·97)	88·28 (16·67)	92·67 (16·96)	< 0·001
UAE, mg/24 h	8·55 (6·02, 15·12)	12·81 (7·92, 30·91)	8·42 (5·99, 14·73)	< 0·001
Biomarkers				
AcAc, μmol/L	37·83 (25·58, 56·85)	41·06 (27·91, 61·37)	37·72 (25·44, 56·58)	0·01
Acetone, μmol/L	19·54 (12·53, 28·88)	23·12 (15·66, 33·20)	19·33 (12·41, 28·53)	< 0·001
Albumin, g/L	44·00 (42·00, 45·00)	44·00 (42·00, 45·75)	44·00 (42·00, 45·00)	0·44
ALP, U/L	66·00 (55·00, 78·00)	72·00 (62·00, 86·00)	65·00 (54·00, 78·00)	< 0·001
ALT, U/L	17·00 (13·00, 24·00)	21·50 (16·00, 32·75)	17·00 (12·00, 23·00)	< 0·001
AST, U/L	22·00 (19·00, 26·00)	24·00 (20·00, 29·00)	22·00 (19·00, 26·00)	< 0·001
Betaine, μmol/L	36·90 (31·00, 43·90)	34·90 (30·20, 42·10)	37·00 (31·10, 44·00)	0·03
BHB, μmol/L	120·18 (91·99, 166·30)	140·55 (111·74, 187·54)	119·12 (91·17, 164·90)	< 0·001
Creatinine, mmol/L	71·00 (62·00, 80·00)	73·00 (63·00, 82·00)	71·00 (62·00, 80·00)	0·05
CRP, mg/L	1·29 (0·60, 2·89)	2·15 (1·13, 3·86)	1·26 (0·59, 2·84)	< 0·001
Ferritine, μg/L	94·00 (46·00, 169·00)	144·0 (77·75, 258·25)	92·00 (45·00, 165·75)	< 0·001
GGT, U/L	23·00 (16·00, 37·00)	37·00 (26·00, 57·00)	23·00 (15·00, 36·00)	< 0·001
GlycA, mmol/L	369.46 (333.22, 413.16)	397.27 (351.68, 437.28)	368.40 (332.21, 411.19)	< 0·001
H1P, μmol/L	3·45 (1·82)	3·13 (1·76)	3·46 (1·83)	0·002
H2P, μmol/L	10·43 (2·81)	11·51 (2·99)	10·37 (2·78)	< 0·001
H3P, μmol/L	3·15 (1·91, 4·42)	2·78 (1·63, 4·05)	3·17 (1·93, 4·44)	< 0·001
H4P, μmol/L	1·70 (1·09, 2·44)	1·32 (0·68, 2·04)	1·71 (1·12, 2·46)	< 0·001
H5P, μmol/L	0·29 (0·03, 0·61)	0·29 (0·06, 0·57)	0·29 (0·03, 0·61)	0·83
H6P, μmol/L	0·62 (0·24, 1·37)	0·35 (0·14, 0·69)	0·64 (0·25, 1·40)	< 0·001
H7P, μmol/L	0·32 (0·12, 0·62)	0·17 (0·05, 0·37)	0·33 (0·13, 0·64)	< 0·001
Hemoglobin, mmol/L	8·51 (0·76)	8·76 (0·79)	8·50 (0·76)	< 0·001
Hematocrit, %	0·41 (0·38, 0·43)	0·42 (0·40, 0·44)	0·41 (0·38, 0·43)	< 0·001
Insulin, mU/L	8·00 (5·70, 11·80)	13·40 (9·00, 20·25)	7·80 (5·60, 11·50)	< 0·001
Isoleucine, μM/L	41·98 (32·57, 52·05)	50·10 (41·09, 62·51)	41·52 (32·29, 51·52)	< 0·001
Leucine, μM/L	124·79 (32·50)	142·49 (35·98)	123·86 (32·05)	< 0·001
MCV, μm3	90·48 (4·64)	90·01 (5·27)	90·50 (4·60)	0·06
TC, mmol/L	5·44 (1·04)	5·63 (1·14)	5·43 (1·03)	0·001
Triglycerides, mmol/L	1·10 (0·80, 1·58)	1·57 (1·07, 2·28)	1·08 (0·79, 1·55)	< 0·001
TMAO, μmol/L	3·20 (1·80, 5·70)	3·50 (1·90, 5·70)	3·20 (1·80, 5·70)	0·43
Transferrin, g/L	2·58 (0·41)	2·65 (0·39)	2·58 (0·41)	0·007
Urea, mmol/L	5·00 (4·30, 6·00)	5·25 (4·50, 6·00)	5·00 (4·20, 6·00)	0·01
Valine, μM/L	203·13 (46·50)	226·56 (51·44)	201·90 (45·90)	< 0·001

Abbreviations: AcAc, Acetoacetate; ALP, alkaline phosphatase; ALT, alanine aminotransferase; AST, aspartate aminotransferase; BMI, body mass index (calculated as weight in kilograms divided by height in meters squared); BHB, beta-hydroxybutyrate; CRP, C-reactive protein; CVD, cardiovascular disease; DBP, diastolic blood pressure; eGFR, estimated glomerular filtration rate; GGT, γ-glutamyltransferase; H1P – H7P: High-density lipoprotein 1–7 particles; IQR, interquartile range; MCV, mean corpuscular volume; SBP, systolic blood pressure; TC, total cholesterol; TMAO, Trimethylamine N-Oxide; UAE, urinary albumin excretion.

Values are shown as mean (SD) or median (25th and the 75th percentile). *P-*values represent the significance of between developers and non-developers of T2D. *P*-values were determined using a 1-way analysis of variance for normally distributed data, Kruskal-Wallis test for skewed distributed data, and χ^2^ test for categorical data.

### 3.2 Biochemical characteristics at baseline

**Fig. 1:**
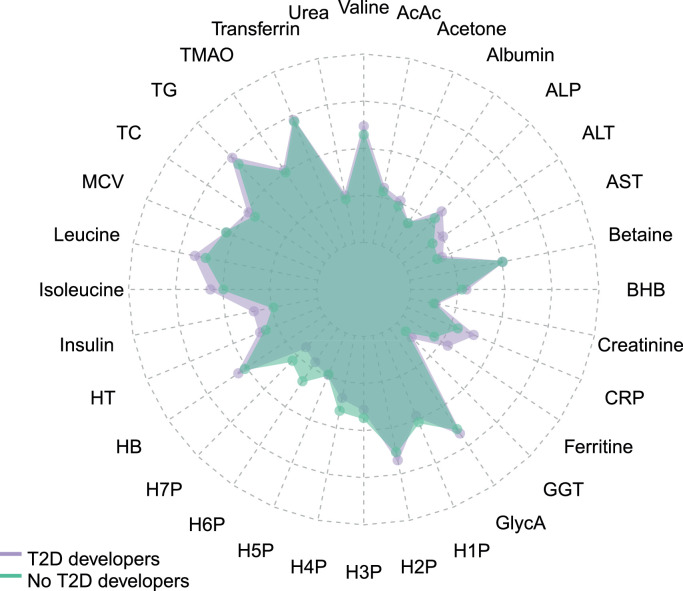
Radarplot showing levels of 32 circulating biomarkers used to calculate the MD in the groups of those developed and did not develop T2D during the follow-up. Biomarkers concentrations are displayed in a scale from 0 to 1, were 0 represents the minimum value and 1 represents the maximum value. Albumin, betaine, creatinine, CRP, ferritin, GlycA, hematocrit, hepatic enzymes, insulin, ketone bodies, triglycerides, TMAO and urea were log transformed to normalize their distributions (*n* = 6247).

Between the groups of participants who developed T2D and those who did not there were marked differences in almost all the biochemical biomarkers, except for plasma albumin, HDL particle 5 (H5P), and mean corpuscular volume (Fig. 1). The following circulating biomarkers were higher in T2D developers: ketone bodies (β-hydroxybutyrate, acetoacetate and acetone), ALP, ALT, AST, creatinine, hsCRP, FPG, ferritin, GGT, GlycA, H2P, hemoglobin, hematocrit, insulin, BCAAs (isoleucine, leucine, valine), total cholesterol, triglycerides, TMAO, transferrin, and urea. Some circulating biomarkers were lower in T2D developers: betaine, H1P, H3P, H4P, H6P and H7P. In participants who developed T2D, UAE was higher, and eGFR was lower (Table 1).

### 3.3 PCA of circulating biomarkers

**Fig. 2:**
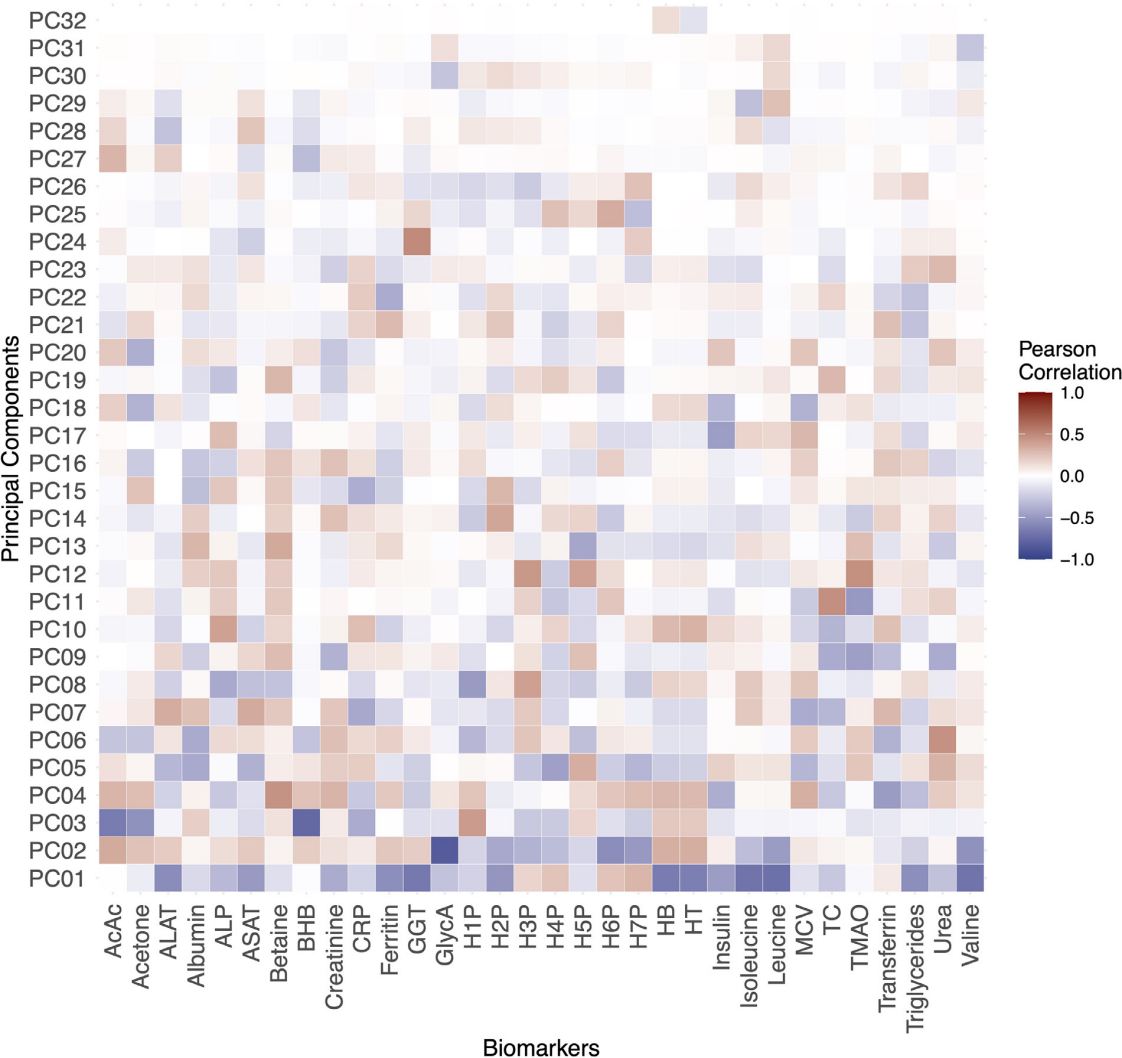
Heatmap showing the correlations between the circulating biomarkers and the PCs (*n* = 6247).

Data of 32 circulating biomarkers: acetoacetate, acetone, albumin, ALP, ALT, AST, betaine, β-hydroxybutyrate (BHB), creatinine, CRP, ferritin, GGT, FPG, H1P, H2P, H3P, H4P, H5P, H6P, H7P, hemoglobin, hematocrit, insulin, isoleucine, leucine, MCV, total cholesterol, triglycerides, TMAO, transferrin, urea, valine were used in the PCA (Supplemental Table 2). The potential biological significance of the PCs is depicted in a heatmap based on pairwise correlations (Fig. 2). The five biomarkers with the highest positive correlation coefficients were: GGT (PC24, *ρ* = 0·49), total cholesterol (PC11, *ρ* = 0·47), TMAO (PC12, *ρ* = 0·46), urea (PC12, *ρ* = 0·46), betaine (PC4, *ρ* = 0·45), (*P*-value for all < 0·0001 [Spearman correlation test]). The five biomarkers with the highest negative correlation coefficients were: GlycA (PC2, *ρ* = -0·83), BHB (PC3, *ρ* = -0·75), leucine (PC1, *ρ* = -0·71), isoleucine (PC1, *ρ* = -0·69), valine (PC1, *ρ* = -0·69) (*P*-value for all < 0·0001 [Spearman correlation test]).).

**Fig. 3:**
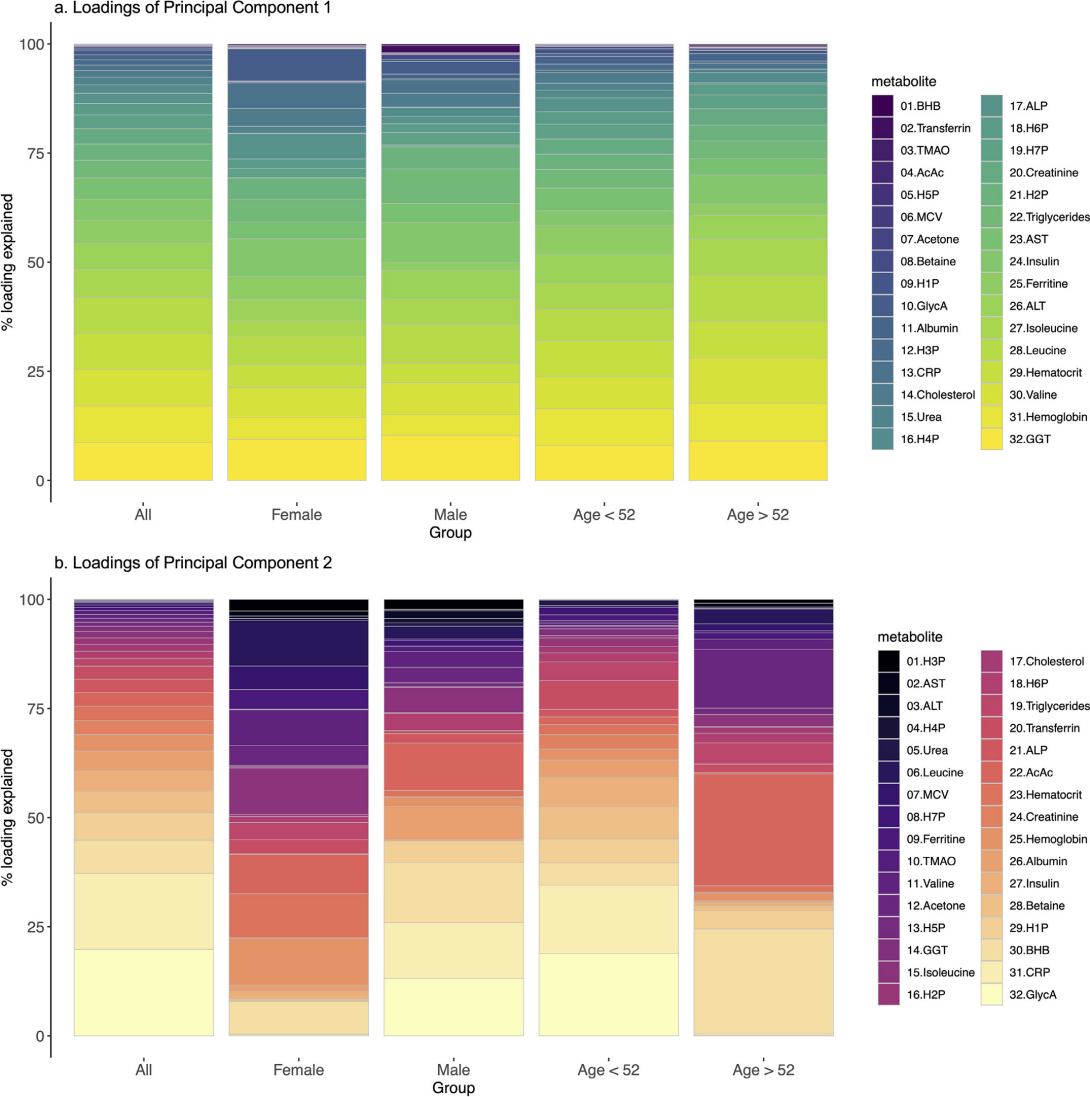
Barplots showing circulating biomarker loadings for the 1st and 2nd PCs across participants subsets (Fig. 3a and b, respectively). Each panel contains the loadings (from left to right) for the whole population (*n* = 6247), females (*n* = 3158), males (*n* = 3089), participants younger than 52 years (*n* = 3146) and older than 52 years (*n* = 3101) (the median age was selected as cutoff point).

Biomarker loadings (the contribution of each biomarker to the PCs) from the first two PCs were congruent with the above reported correlations. The biomarker loadings for the first two PCs are depicted across participants subsets (females, males, participants younger and older than the median age of the cohort, 52 years). Together, the first two PCs captured 26·4% of the total data variation. PC1 captured 16·7% of the variation and PC2 9·7%. In the PC1, the loadings corresponding to BCAA, displayed the biggest differences, being those loadings higher in the group of participants older than 52 years (Δ = 3%), the same was true in the comparison between men and women, being those loadings higher in the group of men (Δ = 2.2%). In the PC2, the loadings corresponding to ketone bodies (acetoacetate, beta-hydroxybutyrate and acetone), displayed the biggest differences, being those loadings higher in the group of participants older than 52 years (Δ = 24%, 19% and 13%, respectively). The PC2 loadings in men and women displayed more differences, being the contribution of hemoglobin and hematocrit smaller in men, compared to women (Δ = 8·6% for hemoglobin and 8·5%, respectively for hematocrit), and the contribution of c-reactive protein and GlycA higher in men compared to women (Δ = 12·8% for c-reactive protein and 12·7% for GlycA) (Fig. 3).

### 3.4 Mahalanobis distance and risk of T2D

During a median follow-up of 7·3 (IQR 6·1–7·3) years, a total of 312 participants developed T2D. Cox proportional hazard regression analyzes were first performed using the 31 MDs calculated based on the subsets of cumulative PCs. All of the MDs were positively associated with an increased risk of T2D (Supplemental Table 3). The association of the MDs with the risk of T2D differed depending on the subset of PCs used to calculate the MDs (Supplemental Table 3). The MD of the subset holding the first 31 PCs was the one with the strongest association, with a HR of 1·72 (95% CI 1·42,2·07), *P* < 0·001, per 1 log-unit increase. The association remained significant after adjustment for age and sex (HR_adj_ 1·70 (95% CI 1·41,2·06), *P* < 0·001), parental history of T2D, plasma glucose, lipid lowering medication and antihypertensive medication (HR_adj_ 1·46 (95% CI 1·19,1·78), *P* < 0·001), BMI (HR_adj_ 1·37 (95% CI 1·11,1·70), *P* = 0·004), and waist circumference (HR_adj_ 1·33 (95% CI 1·07,1·64), *P* = 0·01) (Table 2).

**Table 2:** Prospective associations of MD as continuous and as categorical variable with risk of T2D.

	MD as continuous variable		MD as categorical variable				
	MD per 1 log unit increment		Tertile 1	Tertile 2		Tertile 3	
Participants, *n*	6247		2083	2082		2082	
Events, *n*	312		78	92		142	
	HR (95 % CI)	*P* value		HR (95 % CI)	*P* value	HR (95 % CI)	*P* value
Crude Model	1·72 (1·42,2·07)	<0·001	(ref)	1·15 (0·85,1·56)	0·36	1·99 (1·51,2·63)	<0·001
Model 1	1·70 (1·41,2·06)	<0·001	(ref)	1·18 (0·87,1·60)	0·28	2·00 (1·52,2·64)	<0·001
Model 2	1·46 (1·19,1·78)	<0·001	(ref)	1·10 (0·80,1·50)	0·55	1·49 (1·12,1·98)	0·006
Model 2b	1·42 (1·16,1·75)	<0·001	(ref)	1·03 (0·75,1·41)	0·86	1·44 (1·08,1·92)	0·01
Model 3	1·37 (1·11,1·70)	0·004	(ref)	1·02 (0·75,1·40)	0·88	1·42 (1·10,1·82)	0·01
Model 4	1·33 (1·07,1·64)	0·01	(ref)	1·01 (0·74,1·39)	0·96	1·29 (1·07,1·77)	0·02

Data are presented as hazard ratios (HRs) with 95% confidence intervals (CIs) and *P* values of MD as continuous variable (per 1 log unit increment) and as categorical variable (with the first tertile of MD as reference). MD was calculated using the subset of the first 31 PCs. *P*-values were determined using Cox Proportional-Hazards models.

Model 1. Model adjusted for age and sex.

Model 2. Model 1 plus adjusted for plasma glucose, parental history of T2D, lipid lowering medication and antihypertensive medication.

Model 2b. Model 1 plus adjusted for plasma glucose, parental history of T2D, lipid lowering medication, systolic blood pressure and personal history of CVD.

Model 3. Model 2 plus adjusted for BMI.

Model 4. Model 2 plus adjusted for waist circumference.

Similarly, the analyzes of MD as a categorical variable, using the first tertile as the reference group, showed that the third tertile of MD was associated with a higher risk of T2D in all of the models described above, resulting in a HR of 1·99 (95% CI 1·51,2·63), *P* < 0·001 and a fully adjusted HR_adj_ of 1·42 (95% CI 1·10,1·82), *P* = 0·01 (Table 2). The potential overfitting of the models was assessed with their BIC and their AIC, showing no major difference when using the MD of the subset holding the first 31 PCs in comparison with a smaller number of PCs, i.e., 3 PCs. (BIC: 5166 and 5173, respectively and AIC: 5160 and 5169), respectively), showing that in fact, the model that contains the MD of the subset holding the first 31 PCs performs better. Moreover, the association between risk of T2D and the separated PCs was evaluated. We identified that only the first 6 PCs were associated with the risk of T2D; these associations were less robust than those obtained when using the MD. (Supplemental Table 4)

The association of the MDs with the risk of T2D was also evaluated in men and women, separately. Notably, the association of the MDs with the risk of T2D was greater in women than men for all the PCs subsets (Supplemental Fig. 1.). The MD displaying the strongest association with T2D risk did not correspond to the same PCs subset in men and women. In women, the MD from the first 20 PCs displayed a HR of 2·27 (95% CI 1·74, 2·96), *P* < 0.001; meanwhile, in men, the MD from the 32 PCs displayed a HR of 1·47 (95% CI 1·14, 1·89), *P* < 0.001.

To further asses the benefit of calculating the MD from the PCs instead of using the biomarker raw information, we further evaluate the association of the MD of the 32 circulating biomarkers with the risk of T2D. The MD of the 32 circulating biomarkers was associated with the risk of T2D, showing an unadjusted HR of 1·68 (95% CI 1·46,1·94), *P* < 0·001, per 1 log-unit increase. The association remained after the adjustment for age and sex, (HR_adj_ 1·60 (95% CI 1·38,1·85), *P* < 0·001. Nonetheless, the association did not hold after a full adjustment (HR_adj_ 1·18 (95% CI 0·98,1·40), *P* = 0.07 (Supplemental Table 5). Similarly, the analyzes of MD as a categorical variable, using the first tertile as the reference group, showed that the third tertile of MD was associated with a higher risk of T2D in the crude model, resulting in a HR of 1·61 (95% CI 1·20,2·15), *P* < 0·001; and the association did not hold in the fully adjusted model: HR_adj_ 1·30 (95% CI 0·96,1·76), *P* = 0·09 (Supplemental Table 5).

The comparison of the traditional T2D risk model against the enriched model that included the MD revealed that inclusion of the MD led to a significant improvement in the classification of participants into predicted T2D risk categories, with a NRI of 0·24 (95% CI: 0·18, 0·31) (*P* = 0·001). A category-free version of the NRI (often denoted as NRI > 0) was also computed, resulting in a NRI of 0·74 (95% CI: 0·64, 0·85) (*P* = 0·001).

## 4 Discussion

In this prospective population-based cohort study, we demonstrated the association of MD, a proxy of homeostasis loss, with incidence of T2D. MD is a novel approach for studying changes in collections of biomarkers based on the concept of multivariate statistical distance. In this study, MD measured the abnormality of the whole biomarker profile at baseline in relation to the population mean. The association of MD with increased risk of T2D was independent of age and sex, as well as of anthropometric variables such as BMI and waist circumference.

Along with the development of more efficient high throughput techniques, it is recognized that most of the circulating metabolites are stable over time in healthy subjects, and variations in biomarker profiles could offer a wide-ranging indicator of changes in an individual's health status [42]. The MD of circulating biomarkers has become an alternative means to analyze such variations in high throughput biomarkers and provide a quantifiable proxy of homeostasis loss. Recently, the calculation of MD has been upgraded by replacing the raw biomarker information with PCs [43]. The rationale of replacing the raw biomarkers with PCs is based on the fact that PC analysis could detect underlying processes that might simultaneously regulate the levels of the variables used in the analysis, but may not be directly measurable [44]. Importantly, plasma glucose was not included as part of the PC analysis, in order to prevent the prevailing influence of glucose in assessing degree of homeostasis loss.

In this study, the PC1 loadings corresponding to BCAA were higher in older participants compared to younger participants. This could reflect the already described altered BCAA metabolism in ageing, due to the impaired activity of the mammalian target of rapamycin (mTOR) and mitochondrial dysfunction in ageing [45], characterized by the downregulation of the branched chain aminotransferase 2 [46]. The PC2 loading depicted important differences among the subgroups. This could further correspond to the ageing-induced impairment of ketone body oxidation, regulated by the succinyl-CoA-acetoacetate transferase [47]. Remarkably differences were identified between men and women, in relation to the contribution of inflammatory markers to the PC2 loading, C-reactive protein and GlycA were among the more important contributors to PC2 in men, but its contribution to PC2 in women was remarkably low. Such findings are in line with the reports of a reduced activation and recruitment of leucocytes in women, due to an enhanced response of pro-resolving mediators, including the D-resolvins. Those sex-differences in inflammatory response have been suggested to underly lower incidence of cardiovascular disease in women [48].

The use of PCs accounts for the assessment of the intervariability of biomarkers; previous studies have shown that the PCs could provide an insight of homeostasis dysregulation across multiple physiological systems in patients with chronic diseases, such as T2D and chronic kidney disease [44,49]. The current analyzes revealed that, when MD was calculated based on different cumulative sets of PCs, the association of MD with the risk of T2D was stronger (Supplemental Fig. 1.). This finding reflects the fact that the interactions among biomarkers, better depicts the metabolic changes in the subjects at risk of T2D, rather than the independent effects of individual circulating biomarkers. These results are in line with previous findings reporting similar performance of MD, but in the context of ageing-related outcomes [43]. In our study, the calculation of the MD based on the cumulative set of PCs, instead of the raw biomarker information, helped to depict the association of the MD with the incidence of T2D. Of note, this approach has been previously employed for the investigation of the association of the MD with mortality. Leung et al. computed the MD using the cumulative PCs of 36 circulating biomarkers, and reported a positive association between the MD and the risk of mortality [50]. They found the highest association using the MD based on the first half of PCs, and they reported a critical decline in the association when the last PCs were included in the calculation of the MD. They have argued that the last PCs may represent measurement error or other types of noise [50]. In our analysis, the inclusion of the first 31 PCs represented the strongest association with T2D risk, and the inclusion of the last PC did not further improve the association. Moreover, based on the Akaike Information Criterion and the Bayesian Information Criterion, we found no evidence of overfitting.

The MD was originally developed as a tool to classify subjects based on the joint distribution of different variables and since its application has been restricted to such purposes [51]. It is worth noting that the results of the MD calculation do not merely represent a combination of the biomarkers concentrations, (such as through a PCA), and its association with physiological dysregulation in humans and animals, could remain even when the MD is uncorrelated with its component biomarkers or if such biomarkers are not individually associated with a higher risk of developing a specific clinical outcome [37].

Milot et al., had previously reported the non-significant association between the MD calculated from two different sets of biomarkers [37]. The first set included the concentrations of alanine amino transferase, albumin, albumin/globulin ratio, aspartate amino transferase, calcium, C-reactive protein, Hemoglobin, hematocrit, interleukin 6, iron, and red blood cell count, the MD calculated from this set resulted in a Relative Risk of 1·23 (95% CI 0·77, 2·00), *P* > 0·05. The second set of biomarkers included the plasma concentrations of albumin, basophil count, urea/creatinine ratio, calcium, cholesterol, chloride, creatinine, bilirubin, hematocrit, hemoglobin, osteocalcin, potassium, red blood cell count and sodium, the MD calculated from this set resulted in a Relative Risk of 1·07 (95% CI 0·76, 1·50), *P* > 0·05 [37].

Bearing in mind that T2D has differs in prevalence and consequences between men and women, with women having T2D being at higher risk of complications [52], we considered it of interest to further explore the association of the MDs with the risk of T2D separately in men and women. The association of the MDs with the risk of T2D was greater in women than men (Supplemental Fig. 1.). These results are in line with the findings previously reported by Li et al. about the association between T2D with the MD calculated from 37 biomarkers. In their study, the association was evaluated in two cohorts: Aging in Chianti, (InCHIANTI) and the Women's Health and Aging Study (WHAS); in these, the association of MD with T2D was stronger in the WHAS cohort (OR: 1·22, 95% C.I. 1·08, 1·39), compared to the CHIANTI cohort that included men and women (OR: 1·12, 95% CI 1·00, 1·25) [53]. These results highlight sex differences in the context of T2D pathogenesis. Considering that a higher MD represents a higher degree of homeostasis lost, this finding could signify that once homeostasis regulation is lost, clinical and biochemical risk factors for T2D with sexual dimorphism, such as the higher body fat percentage, fetuin-A, a protein secreted primarily by the liver that regulates insulin signaling [54], neurotensin, a neuro peptide associated with satiety and gut motility [55], sex hormone–binding globulin [56], among others, may exert a major effect. The fact that the MD could better assess the risk of T2D in women could be of pathophysiological relevance, given that typical risk factors are insufficiently able to distinguish the shift from a healthy to an unhealthy phenotype. For instance, in a recent study including more than 90,000 women, 84% of the participants progressed from a from metabolic healthy phenotype to a metabolic unhealthy phenotype, irrespectively of BMI category [57]. Here we reported that the inclusion of MD to a risk model improved the NRI, using predefined T2D risk categories, and also using a category-free version of the NRI (often denoted as NRI > 0). Whereas some authors argue that a category-free NRI may represent a more objective measurement of the improvement in risk prediction because it does not lose information due to categorization [58], other authors have argued that it may overestimate the risk prediction improvement and may not reflect its clinical utility [59].

We acknowledge several strengths of the present study. This study included a large number of participants which allowed us to conduct our analysis with sufficient statistical power. Another strength of the present study is the implementation of robust and validated methods of quantification of novel biomarkers such as betaine, BCAAs, HDL subspecies, and TMAO by means of NMR spectroscopy. To the best of our knowledge, this study is the first to assess the loss of homeostasis with the use of MD of the PCs that contain the information of traditional and novel biomarkers associated with the risk of T2D development.

We are also aware of the limitations of the study. The PREVEND population is mainly comprised of individuals with European ancestry, which limits the generalizability of our findings to persons with different ethnicities. We did not have measurements of biomarkers beyond baseline assessment, which impedes us from evaluating the evolution of the biomarker profiles and therefore the MDs and its association with T2D risk. This fact limits our ability to describe the underlying biological mechanisms. For the same reason, the absence of repeated biomarker measurements prevents us from correcting our analysis for regression dilution. Moreover, the sample size of our study, prevents us from performing a cross-validation analysis. Finally, considering that the MD is a metric that has been proposed to identify outliers in multidimensional datasets [60], it is important to further investigate how the presence of outliers could potentially affect the performance of the MD in the assessment of T2D risk. In this study, a sensitivity analysis conducted in a dataset after outliers removal, the association of the MD with T2D risk remained similar to the association found in the original dataset, being the HRs (1·85 (95% CI 1·47,2·33), *P* < 0·001) and (1·72 (95% CI 1·42,2·07), *P* < 0·001), in the dataset after removal of outliers and in the original dataset, respectively. Further research in this regard is needed.

## 5 Conclusion

This large-scale cohort study demonstrated that higher MD, a novel method for measuring homeostasis loss, is positively associated with incident T2D in both men and women in the general population during extended follow-up. The performance of MD increased by including a larger set of PCs in its calculation, supporting the notion that diminished homeostasis regulation is a result of the interactions among biomarkers, not just their independent effects.

## Declaration of Competing Interest

The authors JLFG, MAG, GN, RPFD and SJLB declare no conflicts of interest. MAC and EG are employees of Labcorp.

## Appendix Supplementary materials

Image, application 1
