# Supplementary material for: Mahalanobis distance, a novel statistical proxy of homeostasis loss is longitudinally associated with risk of type 2 diabetes
Source: eBioMedicine. 2021 Aug 20;71:103550. doi: 10.1016/j.ebiom.2021.103550 (PMC8379628; doi:10.1016/j.ebiom.2021.103550)
Supplement: Supplementary file 1 [file mmc1.docx]

**SUPPLEMENTAL MATERIAL**

**Supplemental Table 1.** STROBE Statement—Checklist of items that should be included in reports of cohort studies

|  | Item No | Recommendation | Page No |
| --- | --- | --- | --- |
| Title and abstract | 1 | (a) Indicate the study’s design with a commonly used term in the title or the abstract | 1 |
|  |  | (b) Provide in the abstract an informative and balanced summary of what was done and what was found | 2 |
| Introduction | | | |
| Background/rationale | 2 | Explain the scientific background and rationale for the investigation being reported | 4 |
| Objectives | 3 | State specific objectives, including any prespecified hypotheses | 4-5 |
| Methods | | | |
| Study design | 4 | Present key elements of study design early in the paper | 5 |
| Setting | 5 | Describe the setting, locations, and relevant dates, including periods of recruitment, exposure, follow-up, and data collection | 6 |
| Participants | 6 | (a) Give the eligibility criteria, and the sources and methods of selection of participants. Describe methods of follow-up | 5 |
|  |  | (b) For matched studies, give matching criteria and number of exposed and unexposed |  |
| Variables | 7 | Clearly define all outcomes, exposures, predictors, potential confounders, and effect modifiers. Give diagnostic criteria, if applicable | 6-8 |
| Data sources/ measurement | 8* | For each variable of interest, give sources of data and details of methods of assessment (measurement). Describe comparability of assessment methods if there is more than one group | 6-8 |
| Bias | 9 | Describe any efforts to address potential sources of bias | 9,10 |
| Study size | 10 | Explain how the study size was arrived at | 5 |
| Quantitative variables | 11 | Explain how quantitative variables were handled in the analyses. If applicable, describe which groupings were chosen and why | 5, 7 |
| Statistical methods | 12 | (a) Describe all statistical methods, including those used to control for confounding | 8-10 |
|  |  | (b) Describe any methods used to examine subgroups and interactions | b.NA |
|  |  | (c) Explain how missing data were addressed | c.5 |
|  |  | (d) If applicable, explain how loss to follow-up was addressed | d.NA |
|  |  | (e) Describe any sensitivity analyses | e.9 |
| Results | | |  |
| Participants | 13* | (a) Report numbers of individuals at each stage of study—eg numbers potentially eligible, examined for eligibility, confirmed eligible, included in the study, completing follow-up, and analysed | 5 |
|  |  | (b) Give reasons for non-participation at each stage | 5 |
|  |  | (c) Consider use of a flow diagram |  |
| Descriptive data | 14* | (a) Give characteristics of study participants (eg demographic, clinical, social) and information on exposures and potential confounders | 20 |
|  |  | (b) Indicate number of participants with missing data for each variable of interest | 5 |
|  |  | (c) Summarise follow-up time (eg, average and total amount) | 12 |
| Outcome data | 15* | Report numbers of outcome events or summary measures over time | 28 |
| Main results | 16 | (a) Give unadjusted estimates and, if applicable, confounder-adjusted estimates and their precision (eg, 95% confidence interval). Make clear which confounders were adjusted for and why they were included | 28 |
|  |  | (b) Report category boundaries when continuous variables were categorized | 28 |
|  |  | (c) If relevant, consider translating estimates of relative risk into absolute risk for a meaningful time period | NA |
| Other analyses | 17 | Report other analyses done—eg analyses of subgroups and interactions, and sensitivity analyses | 13 |
| Discussion |  |  |  |
| Key results | 18 | Summarise key results with reference to study objectives | 14 |
| Limitations | 19 | Discuss limitations of the study, taking into account sources of potential bias or imprecision. Discuss both direction and magnitude of any potential bias | 16 |
| Interpretation | 20 | Give a cautious overall interpretation of results considering objectives, limitations, multiplicity of analyses, results from similar studies, and other relevant evidence | 14-16 |
| Generalisability | 21 | Discuss the generalisability (external validity) of the study results | 16 |
| Other information |  |  |  |
| Funding | 22 | Give the source of funding and the role of the funders for the present study and, if applicable, for the original study on which the present article is based | 2,10, 17 |

*Give information separately for exposed and unexposed groups.

**Supplemental** **Table 2.** Biomarkers included in the calculation of Mahalanobis distance.

| **Biomarker** | **T2D association** | **Most associated PC** |
| --- | --- | --- |
| AcAc | Positive | PC3 |
| Acetone | Positive | PC3 |
| Albumin ˜ | Negative | PC5 |
| ALP ˜ | Positive | PC8 |
| ALT ˜ | Positive | PC1 |
| AST ˜ | Positive | PC1 |
| Betaine | Negative | PC4 |
| BHB | Positive | PC3 |
| Creatinine ˜ | Positive | PC1 |
| CRP ˜ | Positive | PC7 |
| Ferritin ˜ | Positive | PC1 |
| GGT ˜ | Positive | PC1 |
| GlycA | Positive | PC2 |
| H1P ˜ ˜ | Negative | PC8 |
| H2P ˜ ˜ | Positive | PC1 |
| H3P ˜ ˜ | Negative | PC12 |
| H4P ˜ ˜ | Negative | PC5 |
| H5P ˜ ˜ | Positive | PC13 |
| H6P ˜ ˜ | Negative | PC2 |
| H7P ˜ ˜ | Negative | PC2 |
| Hemoglobin ˜ | Positive | PC1 |
| Hematocrit ˜ | Positive | PC1 |
| Insulin | Positive | PC1 |
| Isoleucine | Positive | PC1 |
| Leucine | Positive | PC1 |
| MCV ˜ | Negative | PC7 |
| TC ˜ | Positive | PC11 |
| Triglycerides ˜ | Positive | PC1 |
| TMAO | Positive | PC11 |
| Transferrin ˜ | Positive | PC4 |
| Urea ˜ | Positive | PC6 |
| Valine | Positive | PC1 |

˜ denotes biomarkers used in original report of Mahalanobis distance as proxy of Homeostasis loss (Cohen et al, 2015, DOI 10.1371/journal.pone.0116489). ˜ ˜ Original report included HDL. Abbreviations: AcAc, Acetoacetate; ALP, alkaline phosphatase; ALT, alanine aminotransferase; AST, aspartate aminotransferase; BHB, beta-hydroxybutyrate; CRP, C-reactive protein; GGT, γ-glutamyltransferase; H1P – H7P: High-density lipoprotein 1-7 particles; MCV, mean corpuscular volume; TC, total cholesterol; TMAO, Trimethylamine N-Oxide.

**Supplemental** **Table 3.** Longitudinal associations of MD calculated from different subsets of PCs with risk of T2D.

|  | **HR (95 % CI)** | ***P* value** | **RR_95%_** |
| --- | --- | --- | --- |
| PC1-PC2 | 1·21 (1·12,1·32) | <0·001 | 1.61 (1.33, 2.01) |
| PC1-PC3 | 1·22 (1·10,1·35) | <0·001 | 1.93 (1.37,2.71) |
| PC1-PC4 | 1·28 (1·14,1·44) | <0·001 | 2.45 (1.61,3.77) |
| PC1-PC5 | 1·32 (1·16,1·50) | <0·001 | 2.97 (1.79,4.92) |
| PC1-PC6 | 1·29 (1·13,1·48) | <0·001 | 2.98 (1.68,5.35) |
| PC1-PC7 | 1·25 (1·08,1·45) | <0·001 | 2.82 (1.43,5.64) |
| PC1-PC8 | 1·29 (1·11,1·51) | <0·001 | 3.52 (1.67,7.69) |
| PC1-PC9 | 1·31 (1·11,1·54) | <0·001 | 3.73 (1.66,8.22) |
| PC1-PC10 | 1·39 (1·18,1·64) | <0·001 | 4.87 (2.21,10.79) |
| PC1-PC11 | 1·44 (1·21,1·71) | <0·001 | 5.75 (2.49,13.13) |
| PC1-PC12 | 1·43 (1·20,1·71) | <0·001 | 5.50 (2.38,12.92) |
| PC1-PC13 | 1·44 (1·20,1·72) | <0·001 | 5.69 (2.38,13.28) |
| PC1-PC14 | 1·47 (1·22,1·77) | <0·001 | 6.09 (2.54,14.54) |
| PC1-PC15 | 1·48 (1·23,1·78) | <0·001 | 6.36 (2.65,15.20) |
| PC1-PC16 | 1·48 (1·23,1·79) | <0·001 | 6.61 (2.71,16.54) |
| PC1-PC17 | 1·56 (1·30,1·88) | <0·001 | 8.64 (3.56,21.36) |
| PC1-PC18 | 1·60 (1·33,1·92) | <0·001 | 10.24 (4.10,25.25) |
| PC1-PC19 | 1·59 (1·32,1·92) | <0·001 | 9.83 (3.93,24.92) |
| PC1-PC20 | 1·61 (1·34,1·93) | <0·001 | 10.92 (4.34,27.13) |
| PC1-PC21 | 1·60 (1·34,1·93) | <0·001 | 11.96 (4.68,32.19) |
| PC1-PC22 | 1·58 (1·32,1·90) | <0·001 | 10.89 (4.25,28.51) |
| PC1-PC23 | 1·61 (1·35,1·93) | <0·001 | 12.47 (4.90,35.39) |
| PC1-PC24 | 1·64 (1·36,1·96) | <0·001 | 13.82 (5.11,35.63) |
| PC1-PC25 | 1·67 (1·39,2·00) | <0·001 | 15.38 (5.78,40.22) |
| PC1-PC26 | 1·68 (1·40,2·02) | <0·001 | 17.34 (6.36,47.80) |
| PC1-PC27 | 1·65 (1·37,1·98) | <0·001 | 16.02 (5.72,44.00) |
| PC1-PC28 | 1·64 (1·37,1·98) | <0·001 | 16.36 (5.92,47.44) |
| PC1-PC29 | 1·68 (1·39,2·02) | <0·001 | 18.94 (6.46,53.86) |
| PC1-PC30 | 1·67 (1·39,2·02) | <0·001 | 20.50 (6.95,62.88) |
| PC1-PC31 | 1·72 (1·42,2·07) | <0·001 | 24.65 (7.94,73.68) |
| PC1-PC32 | 1·70 (1·42,2·05) | <0·001 | 23.88 (8.14,73.16) |

Data are presented as unadjusted hazard ratios (HRs) with 95% confidence intervals (CIs) and *P* values. HRs were calculated per 1-unit increase in the log scale. The fourth column presents the Relative Risk difference over 95% (RR_95%_) of the observed MD distribution; this was calculated by subtracting the risk of being in the 97.5^th^ percentile of MD relative to the 2.5^th^ percentile.

**Supplemental** **Table 4.** Prospective associations of different subsets of PCs with risk of T2D.

|  | **HR (95 % CI)** | ***P* value** |
| --- | --- | --- |
| PC1 | 0·74 (0·70,0·77) | <0·001 |
| PC2 | 1·13 (1·06,1·21) | <0·001 |
| PC3 | 0·78 (0·72,0·84) | <0·001 |
| PC4 | 0·81 (0·75,0·87) | <0·001 |
| PC5 | 1·21 (1·11,1·32) | <0·001 |
| PC6 | 0·89 (0·80,0·99) | 0·03 |
| PC7 | 1·01 (0·91,1·11) | 0·87 |
| PC8 | 1·07 (0·97,1·17) | 0·18 |
| PC9 | 1·12 (1·01,1·24) | 0·04 |
| PC10 | 1·08 (0·97,1·20) | 0·17 |
| PC11 | 0·99 (0·89,1·11) | 0·91 |
| PC12 | 1·00 (0·89,1·13) | 0·94 |
| PC13 | 0·93 (0·81,1·05) | 0·25 |
| PC14 | 0·96 (0·84,1·09) | 0·51 |
| PC15 | 1·11 (0·96,1·29) | 0·15 |
| PC16 | 0·89 (0·77,1·03) | 0·11 |
| PC17 | 0·88 (0·78,0·99) | 0·04 |
| PC18 | 0·93 (0·80,1·08) | 0·33 |
| PC19 | 1·05 (0·91,1·21) | 0·48 |
| PC20 | 0·98 (0·83,1·16) | 0·83 |
| PC21 | 1·28 (1·07,1·52) | 0·01 |
| PC22 | 1·04 (0·86,1·24) | 0·71 |
| PC23 | 0·86 (0·72,1·02) | 0·08 |
| PC24 | 1·06 (0·90,1·25) | 0·46 |
| PC25 | 1·12 (0·95,1·32) | 0·18 |
| PC26 | 1·07 (0·91,1·26) | 0·43 |
| PC27 | 1·02 (0·82,1·26) | 0·86 |
| PC28 | 0·84 (0·68,1·04) | 0·11 |
| PC29 | 1·15 (0·94,1·42) | 0·17 |
| PC30 | 1·13 (0·88,1·46) | 0·35 |
| PC31 | 0·81 (0·61,1·06) | 0·13 |
| PC32 | 0·75 (0·49,1·15) | 0·19 |

**Supplemental** **Table 5.** Prospective associations of MD calculated from 32 circulating biomarkers concentrations with risk of T2D.

|  | **MD as continuous variable** | | **MD as categorical  variable** | | | | |
| --- | --- | --- | --- | --- | --- | --- | --- |
|  | **MD per 1 log unit increment** | | **Tertile 1** | **Tertile 2** | | **Tertile 3** | |
| Participants, n | 6247 | | 2083 | 2082 | | 2082 | |
| Events, n | 312 | | 70 | 92 | | 150 | |
|  | **HR (95 % CI)** | ***P* value** |  | **HR (95 % CI)** | ***P* value** | **HR (95 % CI)** | ***P* value** |
| Crude Model | 1·68 (1·46,1·94) | <0·001 | (ref) | 1·26 (0·91,1·73) | 0·15 | 1·61 (1·20,2·15) | 0·001 |
| Model 1 | 1·60 (1·38,1·85) | <0·001 | (ref) | 1·21 (0·88,1·67) | 0·23 | 1·51 (1·13,2·04) | 0·005 |
| Model 2 | 1·18 (1·00,1·40) | 0·05 | (ref) | 1·21 (0·88,1·67) | 0·24 | 1·45 (1·07,1·96) | 0·01 |
| Model 3 | 1·18 (0·99,1·40) | 0·06 | (ref) | 1·12 (0·81,1·55) | 0·50 | 1·31 (1·97,1·77) | 0·08 |
| Model 4 | 1·18 (0·98,1·40) | 0·07 | (ref) | 1·11 (0·80,1·54) | 0·51 | 1·30 (0·96,1·76) | 0·09 |

Data are presented as hazard ratios (HRs) with 95% confidence intervals (CIs) and *P* values of MD as continuous variable (per 1 log unit increment) and as categorical variable (with the first tertile of MD as reference). MD was calculated using the circulating concentrations of 32 biomarkers.

Model 1. Model adjusted for age and sex

Model 2. Model 1 adjusted for plasma glucose, parental history of T2D, lipid lowering medication and antihypertensive medication.

Model 3. Model 2 adjusted for BMI.

Model 4. Model 2 adjusted for waist circumference.

**Supplementary Fig.1.**

**
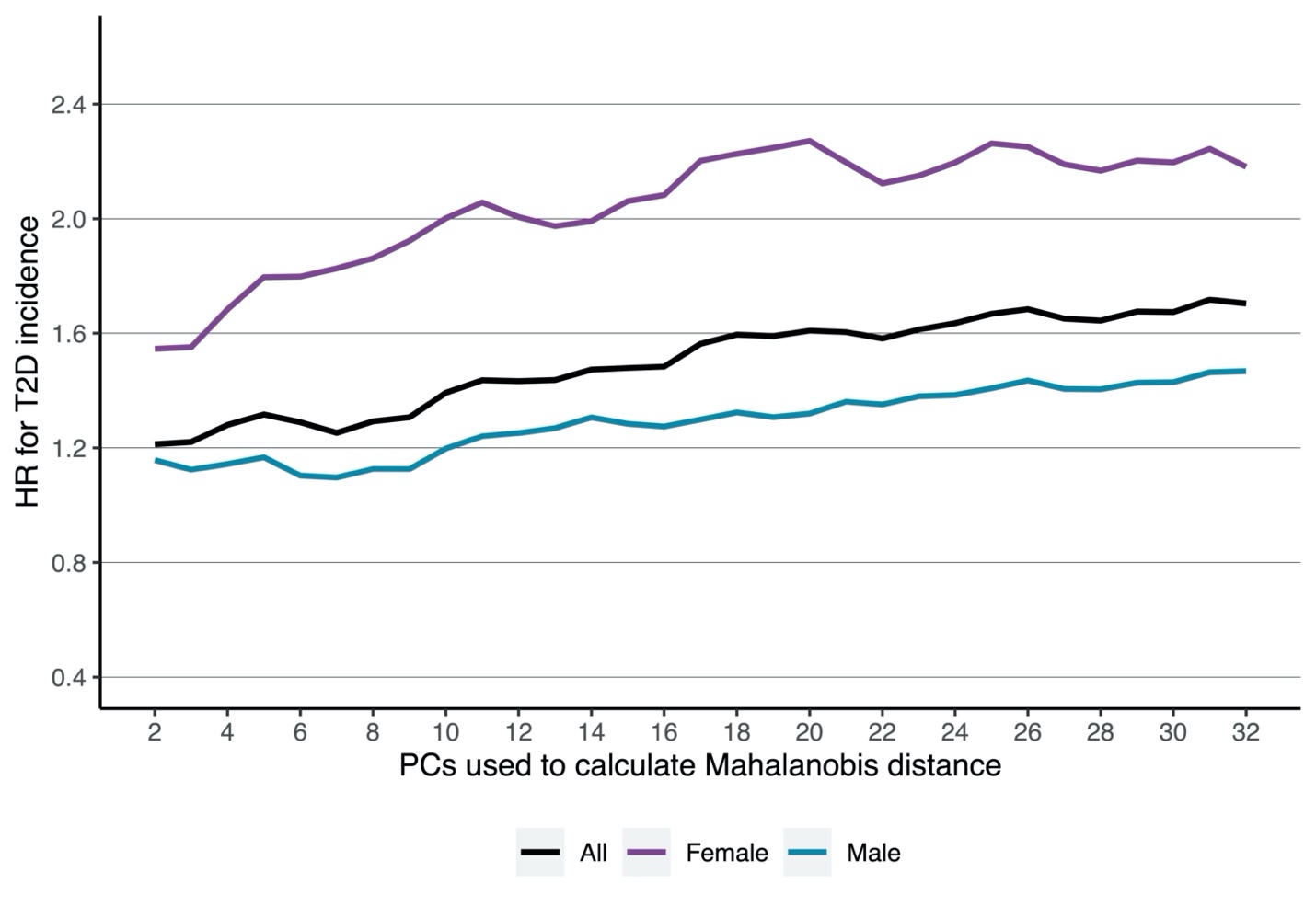
**

Supplementary Fig.1. Plot showing the association of MD and risk of T2D. HRs for incidence of T2D (*y*-axis), were obtained for 32 different MDs, calculated from cumulative subsets of PCs (*x*-axis). HRs were computed for the whole population (black line), men (blue) and women (purple).

**Supplementary methods.**

TMAO, betaine, BCAA and ketone body concentrations were measured in EDTA-anticoagulated plasma samples using a Vantera® Clinical Analyzer (LabCorp, Morrisville, NC), a fully automated, high-throughput, 400 MHz proton (^1^H) nuclear magnetic resonance (NMR) spectroscopy platform. TMAO was quantified from one-dimensional (1D) proton (^1^H) Carr-Purcell-Meiboom-Gill (CPMG) spectra by spectral deconvolution algorithm as previously described (1,2). The TMAO assay has intra- and inter-assay coefficients of variation (CV%) range from 4.3–10.3% and 9.8–14.5%, respectively, and a limit of quantitation of 3.3 μM (2).

The validation of the use of NMR for quantification of BCAAs has been previously described by our group (3). In brief, coefficients of variation for inter- and intraassay precision ranged from 1.8% to 6.0%, 1.7% to 5.4%, 4.4% to 9.1%, and 8.8% to 21.3%, for total BCAAs, valine, leucine, and isoleucine, respectively. BCAAs quantified from the same samples using NMR and LC-MS/MS were highly correlated (*r* = 0.97, 0.95 and 0.90 for valine, leucine, and isoleucine) (3).

For determining ketone body concentrations, a method comparison study was performed comparing quantification by NMR to platforms commonly used, i.e LC/MS/MS for β-hydroxybutyrate and acetoacetate and GC/MS for acetone. A comparison of plasma

concentrations using the comparator platforms correlated well by Deming regression with R^2^ values of 0.996, 0.994 and 0.994 for β-hydroxybutyrate, acetoacetate and acetone, respectively. The limits of quantification were calculated to be 65.0, 45.0, 26.3 and 19.7 µM for total ketone bodies, β-hydroxybutyrate, acetoacetate and acetone, respectively. For β-hydroxybutyrate, acetoacetate and acetone coefficients of variation for intra-assay and inter-assay precision were 1.3%–9.3%, 3.1%–7.7%, and 3.8%–9.1%, respectively. A more detailed description of the data acquisition and method validation has been previously reported (4).

**References**.

1.         Garcia E, Osté MCJ, Bennett DW, Jeyarajah EJ, Shalaurova I, Gruppen EG, Hazen SL, Otvos JD, Bakker SJL, Dullaart RPF, Connelly MA. High Betaine, a Trimethylamine N-Oxide Related Metabolite, Is Prospectively Associated with Low Future Risk of Type 2 Diabetes Mellitus in the PREVEND Study. *Journal of Clinical Medicine* 2019;8(11):1813.

2.         Garcia E, Wolak-Dinsmore J, Wang Z, Li XS, Bennett DW, Connelly MA, Otvos JD, Hazen SL, Jeyarajah EJ. NMR quantification of trimethylamine-N-oxide in human serum and plasma in the clinical laboratory setting. *Clinical Biochemistry* 2017;50(16–17):947–955.

3.         Connelly MA, Wolak-Dinsmore J, Dullaart RPF. Branched Chain Amino Acids Are Associated with Insulin Resistance Independent of Leptin and Adiponectin in Subjects with Varying Degrees of Glucose Tolerance. *Metabolic Syndrome and Related Disorders* 2017;15(4):183–186.

4.         Garcia E, Shalaurova I, Matyus SP, Oskardmay DN, Otvos JD, Dullaart RPF, Connelly MA. Ketone Bodies Are Mildly Elevated in Subjects with Type 2 Diabetes Mellitus and Are Inversely Associated with Insulin Resistance as Measured by the Lipoprotein Insulin Resistance Index. *Journal of Clinical Medicine* 2020;9(2):321.
